# On the Brain of a Bushwoman

**Published:** 1864-02

**Authors:** John Marshall

**Affiliations:** Surgeon to University College Hospital


### ON THE BRAIN OF A BUSHWOMAN.

By JOHN MARSHALL, F.R.S., Surgeon to University College Hospital.

The Bushwoman was aged, and about 5 feet high—unusual for her race.

The form of cranium is a long narrow ovoid—less dolichocephalic, however, than the negro skull; the face is high-cheeked, and the nose very small and flattened. The frontal sinuses are absent, and the walls of the cranium are thick—so thick that its internal capacity is less than would be expected from its outward form and size, being equal to 35 oz. av. of water, or 60.64 cubic inches, which, for the height of the Bushwoman's body, is decidedly, but not very small.

The actual weight of the preserved encephalon proved to be 21.77 oz. av., which would probably represent, as the author shows, 31.5 oz. for the weight of the recent brain enclosed in its membranes. Allowance being made for the height of the body, this is less by 8.5 oz. than the average weight of the brains of European females of the same age, as estimated from the Tables of Dr. Boyd, published in the Philosophical Transactions for 1861.

The cerebrum proper probably weighed, in its recent state,

27.25 oz., the cerebellum 3.45 oz., and the pons with the medulla oblongata .8 oz.

The ratio of the cerebrum to the cerebellum was as usual, 7.7 to 1; that of the cerebrum to the body was probably as 1 to 52, and that of the cerebellum to the body as 1 to 418, instead of the usual ratios of 1 to 41, and 1 to 328.

An examination of the general form of the cerebrum shows that it is small, but long—defective in width, and especially in height. Its outlines and surfaces are angular and flat, instead of rounded and full. The frontal region is very narrow, shallow, much excavated below, and compressed latterally near the entrance of the Sylvian fissure. The parietal region is low, but prominent latterally; the occipital region is long, but defective in height; and the temporal region is long, but narrow.

The cerebrum overlaps the cerebellum by .5 inch, which is as great an absolute overlap as is usual in European brains, but less relatively to the length of the brain, which is very long in the Bushwoman.

The fissures, lobes, and convolutions are then described at length, and compared with those of the ordinary European brain, with those of the Hottentot Venus's brain figured by Gratiolet, and with those of the young Chimpanzee. It is impossible to give in an abstract even an outline of the facts recorded in this part of the paper.

The general result of the inquiry is to show that the fissures are rather more complex than in the brain of the Hottentot Venus, but much less so than in the European. They are rather more complex on the left than on the right side of the brain. They are widely separated from those of the Ape's brain.

The author concludes—1. That all the convolutions proper to man are present, but, as compared with the European brain, are much more simple, and less marked with secondary sulci. The greatest deficiency is in the occipital and orbital convolutions.

2. That the convolutions, taken generally, are rather more complex than those represented in Gratiolet's figure of the Hottentot Venus's brain, which may be partly due to the obliteration of details in the latter during its long period of preservation.

3 & 4. That the resemblance between the Bushwoman's brain and the Hottentot Venus's brain is sufficient to justify the conclusion that the latter was not an idiot, or a defectively developed individual; but both brains, as compared with the European, have an infantile simplicity, characteristic partly of sex, but chiefly of race.

5. That the convolutions being more simple, can be more easily traced and compared on the two sides than usual, but still show abundant evidences of the asymmetry characteristic of man.

6. That there is a greater difference between the Bushwoman's cerebrum and the highest Ape's cerebrum than between it and the European cerebrum; but a less specific difference between it and the European than between the Chimpanzee and the Orang; and, of course, much less than between the highest and lowest Quadrumanous brains. There is, however, less difference between the Bushwoman and the highest Ape than between the latter and the lowest Quadrumanous animal.

7. The general results, the author thinks, justify the expectation that characteristic differences of degree of cerebral development may hereafter be found in the several leading races of mankind.

The author then proceeds to describe the color and relative proportions of the grey and white substance, the commissures, ventricles, and ganglionic masses.

The commissural fibres of the corpus callosum are very deficient in the Bushwoman; and the other commissures are also small. The body and anterior cornu of the lateral ventricle are also small; but the potserior cornu and its contained parts are very large.

In the cerebellum, the median parts appear to be somewhat less developed than the hemispheres. Its transverse commissural fibres are more largely developed than the same system of fibres in the European brain; the Chimpanzee standing, in this respect, still lower. The laminæ of the cerebellum are even more numerous than in the European specimen with which the Bushwoman's brain was compared. The cerebellum seems to be more perfectly developed than the cerebrum.—*Boston Medical and Surgical Journal.*

